# Prevalence and predictors of depressive symptoms among people living with HIV in Pakistan; country-wide secondary data analysis from National Stigma Index Study

**DOI:** 10.1371/journal.pgph.0003882

**Published:** 2024-11-04

**Authors:** Usman Ali, Umar Riaz, Heather Doyle, Asghar Satti, Nashmia Mahmood, Summayyah Rasheed, Kalsoom Zahra

**Affiliations:** 1 Department of Management, Association of People Living with HIV, Pakistan; 2 Program Management Unit, United Nations Development Program (UNDP), Pakistan; 3 Public Health Consultant, Pakistan; South African Medical Research Council, SOUTH AFRICA

## Abstract

The objective of this analysis is to identify the prevalence of depressive symptoms and its predictors in the national cohort of people living with HIV (PLHIV) in Pakistan. This is a secondary data analysis of the National Stigma Index Study 2.0. We screened PLHIV in the Stigma Index study for depressive symptoms using the Urdu version of the Patient Health Questionnaire (PHQ)-9. We used stepwise multiple linear regression to identify predictors of depressive symptoms. We also explored the moderating effect of stigma faced by PLHIVs while accessing HIV health services on depressive symptoms. Data was analyzed using the Statistical Package for Social Sciences Version 26 and PROCESS MACRO Version 4.2. A total of 1,497 PLHIV participated in the original study. Based on the PHQ-9 depressive symptom categories, 39.89% had no depressive symptoms, 24.42% had mild depressive symptoms, 16.89% had moderate depressive symptoms, 10.17% had moderately severe depressive symptoms, and 8.61% had severe depressive symptoms. Results of multiple linear regression show that being worried to meet basic life needs such as food and shelter in last 12 months (2.188, 95% Confidence interval 3.98–5.68, p < .01), female sex (3.599, 95% CI 2.703–4.49, p < .01), substance use (31.33, 95% CI 2.379–3.88, p < .01), being employed (-1.627, 95% CI -2.36 to -.88, p < .01), being recruited through limited chain referral as opposed to recruitment from HIV service delivery sites (-2.147, 95% CI -3.41 to -.88, p< .01), and doing sex work (1.143, 95% CI .225–2.061, p < .01) were significant predictors of depressive symptoms. There is a high prevalence of depressive symptoms among PLHIV in Pakistan. Inability to meet basic life needs, female sex, substance use, employment, and facing stigma in the healthcare setting were predictors of depression. There is a need of socioeconomic empowerment, stigma reduction in healthcare settings, and a robust screening program for depressive symptoms for PLHIV community in the country.

## Introduction

Human immunodeficiency virus (HIV) has caused a global epidemic since the 1980s. HIV is transmitted through contaminated needles and unprotected sexual intercourse [1]. The risk of HIV is higher among certain populations compared to the general population. These populations are called key populations. They include people who use substance for recreation, men who have sex with men (MSM), transgender people (TG), sex workers (SW) and prisoners [2]. The former four key populations are criminalized in various countries such as Pakistan. In addition, cultural and religious stigma exists against these key populations. The lack of treatment in the early days of HIV epidemic and multiple severe infections among people living with HIV (PLHIV) compounded the stigma against key populations. This stigma adds to various challenges in life of key populations and PLHIV [3].

In Pakistan, about 270,000 people are living with HIV, with a country prevalence of 0.1% [4] HIV is a concentrated epidemic among key populations in the country. According to the last round of Integrated Biological and Behavioral Surveillance (IBBS 2017), 38.2% of people who inject substance, 7.1% of transgender people (TG), 5.4% of men who have sex with men (MSM), and 2.2% of female sex workers (FSW) are living with HIV [5]. In addition to the concentrated epidemic in key populations, there are various outbreaks of HIV reported in the general population. These are attributed to poor infection prevention and control practices. There are a total of eight outbreaks reported in the country in various districts, affecting mostly the rural communities [6].

HIV key populations are stigmatized and criminalized in Pakistan. According to Section 377 of the Pakistan Penal Code, sexual activity between consenting adults is a cognizable offense with the death penalty. The Hudood Ordinance also penalizes sex outside wedlock and sex work [7]. In addition, illicit substance possession of any quantity is penalized under Section 9 of the Control of Substance and Narcotics Act 1997. According to IBBS 2017, 63.8% of people who inject substance, 31.3% of MSM, 52% of TG and 35.6% of female sex workers (FSWs) also reported being discriminated against [5]. However, the sample constituted of key populations, regardless of HIV status. The most recent HIV Stigma Index Study recruited PLHIV to gauge stigma in various dimensions. According to the survey, 4.32% of PLHIV faced social exclusion. Due to perceived social stigma, 31.78% of all PLHIV have avoided social gatherings [3]. The key population segregated data show that 1.21% of MSM, 5.81% of TG, 10.85% of people who use substance for recreation, and 2.21% of sex workers (SW) faced social exclusion. While 31.83% MSM, 37.93% of TG, 44.95% of people who use substance for recreation and 35.98% of SW chose to avoid social gatherings due to perceived social stigma. Among PLHIV, 33.71% stated that HIV negatively affected their self-confidence, and 35.16% stated that HIV negatively affected their self-respect [3].

According to a recent systematic review, the overall prevalence of common mental health conditions in PLHIV is 28–62%. Among common mental health conditions depression is the most prevalent in PLHIV followed by generalized anxiety disorder [8]. The impact of mental illness on PLHIV is not limited to poor quality of life but also has an impact on adherence to antiretroviral treatment [8]. Depression in PLHIV can be attributed to variety of factors. Substance use is an established risk factor for depressive disorders [9]. In addition, depression is associated with similar chronic illnesses such as diabetes, hypertension etc [10]. Depressive symptoms may be attributed to unique stressors that people from key populations face. Meyer proposed an influential model called ‘minority stress theory’. According to the theory, in addition to general stress faced by people from sexual and gender minorities, face unique stress which he called the minority stress. This includes distal stressors such as social disadvantage due to sociocultural stigma, laws and policies, as well as proximal stressors such as everyday discrimination faced by transgender people and men who have sex with men. This stress can translate into negative health outcomes including mental health conditions [11, 12]. Tao and colleagues have also demonstrated that external HIV stigma and internalized stigma increase odds of depression among PLHIV by 4% and 9% respectively [13].

The mental health of PLHIV in Pakistan has not been systematically studied. There is a dearth of research with only two researches on mental health of PLHIV in Pakistan [14, 15]. Both these studies recruited study samples from HIV treatment centers and were single-center studies. Junaid et al. reported a prevalence of 32.2% among PLHIV from Lahore whereas Ahmed et al. reported a prevalence of 89.9% of depression and 80.3% of anxiety [14, 15]. The current study is from a national cohort and the first to report prevalence and predictors of depressive symptoms among PLHIV across Pakistan.

## Materials and methods

### Ethics statement

The principal study (National HIV Stigma Index 2.0) was approved by National Bioethics Committee (Reference No.4-87/NBCR-987/23/411). Informed consent was obtained from all participants included in the study.

### Study design and setting

This is a secondary data analysis of a cross-sectional survey, National HIV Stigma Index Study 2.0. HIV Stigma Index is a global tool for gauging the stigma faced by PLHIV due to HIV and key population status. More details of HIV Stigma Index can be found at www.stigmaindex.org. Data collection for primary study was conducted from October to December 2023. The study was conducted throughout Pakistan using non-probability sampling. Only consenting participants were included in the study. The participants were recruited using venue-based sampling from HIV service delivery sites. These include community-based HIV prevention sites and HIV treatment centers. Sites for recruiting key populations were prioritized based on the HIV prevalence among key population as estimated in the Integrated Biological and Behavioral Surveillance 2016–17 [5]. These cities were included in order of priority, for example, for Punjab, the top four prioritized cities were included and so forth for other provinces. For the non-key population PLHIV in Punjab and Sindh, we resorted to the sites where HIV outbreaks were reported as cited in the report on the Larkana HIV outbreak. These areas of outbreak are: Larkana (3 outbreaks), Sargodha (2 outbreaks) and Hyderabad, Jalapur Jattan, Chiniot each with one outbreak [6]. We selected all these sites. These are rural areas; thus, the sample included the rural population. We also included Dera Ghazi Khan and Sukkur which reported the highest number of PLHIV according to program data outside Lahore and Karachi (the largest cities of the province). Since there are no outbreaks reported from Khyberpakhtunkhawa (KPK), Islamabad capital territory (ICT) and Balochistan, we included the cities which had the highest number of PLHIV registered at HIV treatment centers. These were Peshawar and Bannu for KPK, and Quetta and Turbat for Baluchistan. (Source: Program data, United Nations Development Program, UNDP). The other method of participant recruitment was limited chain referral (LCR) which involved community referrals. This strategy was used to target key populations and PLHIV who were not currently accessing care and treatment. These populations were reached through PLHIV who participated in the survey through the HIV service delivery sites. PLHIV who completed the survey were given data collectors contact numbers. Potential participants referred by those interviewed can contact the data collectors. The data collectors screened them to ensure that they met the eligibility criteria for the study. Eligible potential participants who provided written consent were thus included. Confidentiality, safety and privacy were ensured by allocating codes to participants filled pro forma and access to data (electronic or hard copy) was limited to authorized personnel among the study staff.

The sample size was divided into key populations based on PLHIV proportions mentioned in the United Nations Joint Program on AIDS (UNAIDS) country snapshot for Pakistan 2023. According to the report, 24% of the PLHIV pool in the country consists of MSM, 25% of people who use substance, 2% of female sex workers and TG and 47% of the general population. We have ensured people who use substance and MSM have the same proportion and increased the number of FSW, TG in the sample again for obtaining meaningful data [16]. We also included 50 (non-female) sex workers in our sample. The criteria for inclusion in the study were that the person must know their HIV status for at least 12 months, must be 18 years of age or older and must be able to provide consent. We excluded PLHIV who were employed at any service delivery site from the study or those who have come to, know their HIV status in the last 12 months.

### Study instrument

The Patient Health Questionnaire (PHQ-9) was used to screen for depressive symptoms. PHQ-9 is a nine-item questionnaire rated on a four-point Likert scale of four. The scale is a screening tool for major depressive disorder (MDD). The PHQ-9 is an open-access questionnaire that can be used for screening through self-administration or administered by an interviewer. The PHQ-9 requires minimal training for administration. Urdu translation has been validated and readily available. The Urdu translation of PHQ-9 has been validated for people living with chronic diseases including HIV [17–19]. In PLHIV PHQ-9 has good internal consistency among PLHIV with a Cronbach’s alpha of .759 [19]. The scale has standardized composite score; 0–4 no or minimal depressive symptoms, 5–9 mild depressive symptoms, 10–14 moderate depressive symptoms, 15–19 moderately severe depressive symptoms, and 20–27 severe depressive symptoms [18]. Gholizadeh et al. showed that the for Urdu version of the PHQ-9 using a cut-off of ≥ 6 yielded a sensitivity of 76.1%, specificity of 75.9%, a positive predictive value of 34%, and a negative predictive value of 95.1% [17].

Stigma was measured in two dimensions, i.e., social stigma and stigma while accessing HIV health services. Both variables were coded as categorical. Social stigma is defined if participants have faced any of the following in the last 12 months due to HIV status: excluded from family gatherings, religious activities, family activities, family members, or other people have gossiped about their HIV status, if they were verbally or physically harassed, blackmailed, refused employment, or lost source of income or job description was changed solely due to HIV status or if the family members had faced stigma due to their HIV status. Likewise, stigma while accessing HIV health services is defined when participants have faced any of the following in the last 12 months: denial of health services, advised not to have sex, talked badly or gossiped about in a healthcare setting, verbally or physically abused in a healthcare setting, avoidance of physical contact or taking extra precautions (wearing double gloves etc.) during clinical encounters and disclosure of HIV status without consent to others.

### Statistical analysis

Frequencies and percentages were computed for categorical variables. Comparison of sociodemographic characteristics was done between those screened positive for depressive symptoms with a cut of more than equal to and more than 6 and those without depressive symptoms with a score of ≤ 5 on PHQ-9. The chi-square was used to ascertain group differences. Factors significantly associated with depressive symptoms and having a correlation of 0.1 or more were included in the stepwise multiple linear regression. Assumptions for multiple linear regression were checked as described in the literature [20]. We also checked the moderating effect of stigma while accessing HIV health services on association of participant recruitment method and depressive symptoms. Data was analyzed using the Statistical Package for Social Sciences (SPSS) version 26.0 and PROCESS Macro Version 4.2 and reported.

## Results

**Fig 1:**
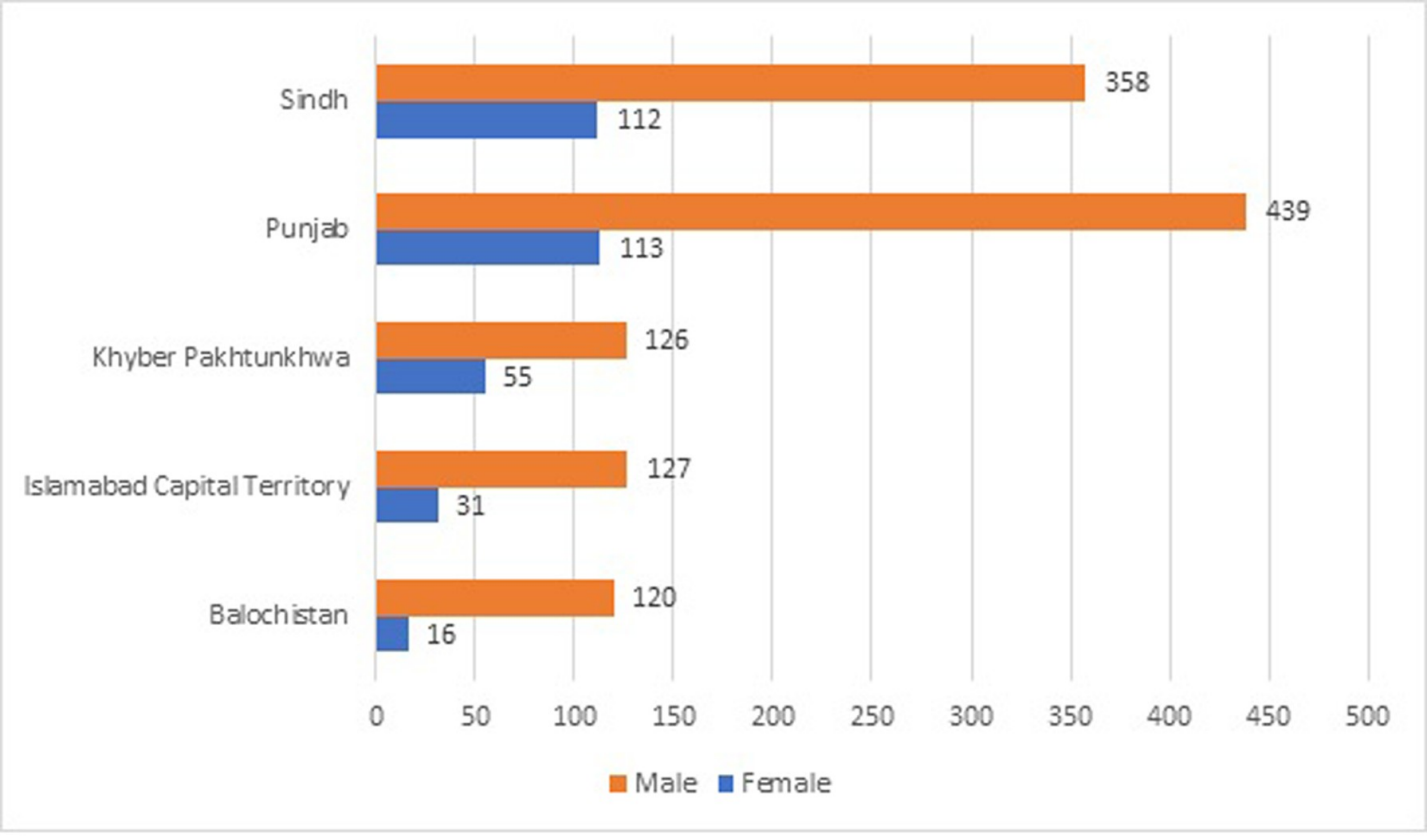
Geographical units’ contribution in a sample of National HIV Stigma Index 2.0.

A total of 1,497 participants were recruited from all geographical units of the country. About 1170 (78.15%) were assigned the male sex at birth and 327 (21.84%) were assigned female sex at birth. The distribution of sample from national geographical units is shown in Fig 1.

A comparison of characteristics of the study sample, whether they had depressive symptoms or not and results of the chi-square test are given in Table 1. Group differences were significant between two groups for biological sex, gender, sexual partner status, employment status, being unable to meet life needs, being a sex worker, having sex with men, identifying as bisexual, substance use, and recruitment strategy.

**Table 1:** Comparison of characteristics of study participants among those with depressive symptoms compared to those without depressive symptoms (PHQ-9 cut-off ≥ 6).

Serial No.	Variable	No depressive symptoms	With Depressive symptoms	p-value
**1.**	Biological Sex			.000
	Male	634 (42.46%)	534 (35.77%)	
	Female	118 (7.90%)	207 (13.86%)	
**2.**	Gender			
	Man	538 (36.16%)	440 (29.57%)	.000
	Woman	119 (8.00%)	206 (13.84%)	
	Transgender	89 (5.98%)	96 (6.45%)	
**3.**	Partnered			.011
	Yes	461 (30.92%)	502 (33.67%)	
	No	289 (19.38%)	239 (16.03%)	
**4.**	Partner HIV status			.459
	Living with HIV	345 (35.60%)	362 (37.36%)	
	Not living with HIV or do not know status	121 (12.49%)	141 (14.55%)	
**5.**	Are you currently a student?			.635
	Yes	61 (4.10%)	65 (4.37%)	
	No	689 (46.33%)	672 (45.19%)	
**6.**	Are you employed			.000
	Yes	480 (32.15%)	352 (23.58%)	
	No	271 (18.15%)	390 (26.12%)	
**7.**	In the last 12 months, how often have you been unable to meet basic needs (e.g., food, shelter, or clothing)?			.000
	Never	264 (17.77%)	113 (7.60%)	
	Some of the time	295 (19.85%)	358 (24.09%)	
	Most of the time	187 (12.58%)	269 (18.10%)	
**8.**	Do you identify as gay or as men who have sex with men?			.000
	Yes	147 (12.59%)	189 (16.18%)	
	No	487 (41.70%)	345 (29.54%)	
**9.**	Do you identify as bisexual or have sex with both men and women?			.023
	Yes	106 (7.09%)	76 (5.08%)	
	No	647 (43.25%)	667 (44.59%)	
**10.**	Do you do sex work?			.000
	Yes	97 (6.48%)	148 (9.89%)	
	No	656 (43.85%)	595 (39.77%)	
**11.**	Do you regularly use or inject any substance for recreation?			.000
	Yes	164 (10.96%)	262 (17.51%)	
	No	589 (39.37%)	481 (32.15%)	
**12.**	Recruited through			.000
	HIV service delivery site	661 (44.36%)	707 (47.45%)	
	Limited Chain referral	88 (5.91%)	34 (2.28%)	

**Fig 2:**
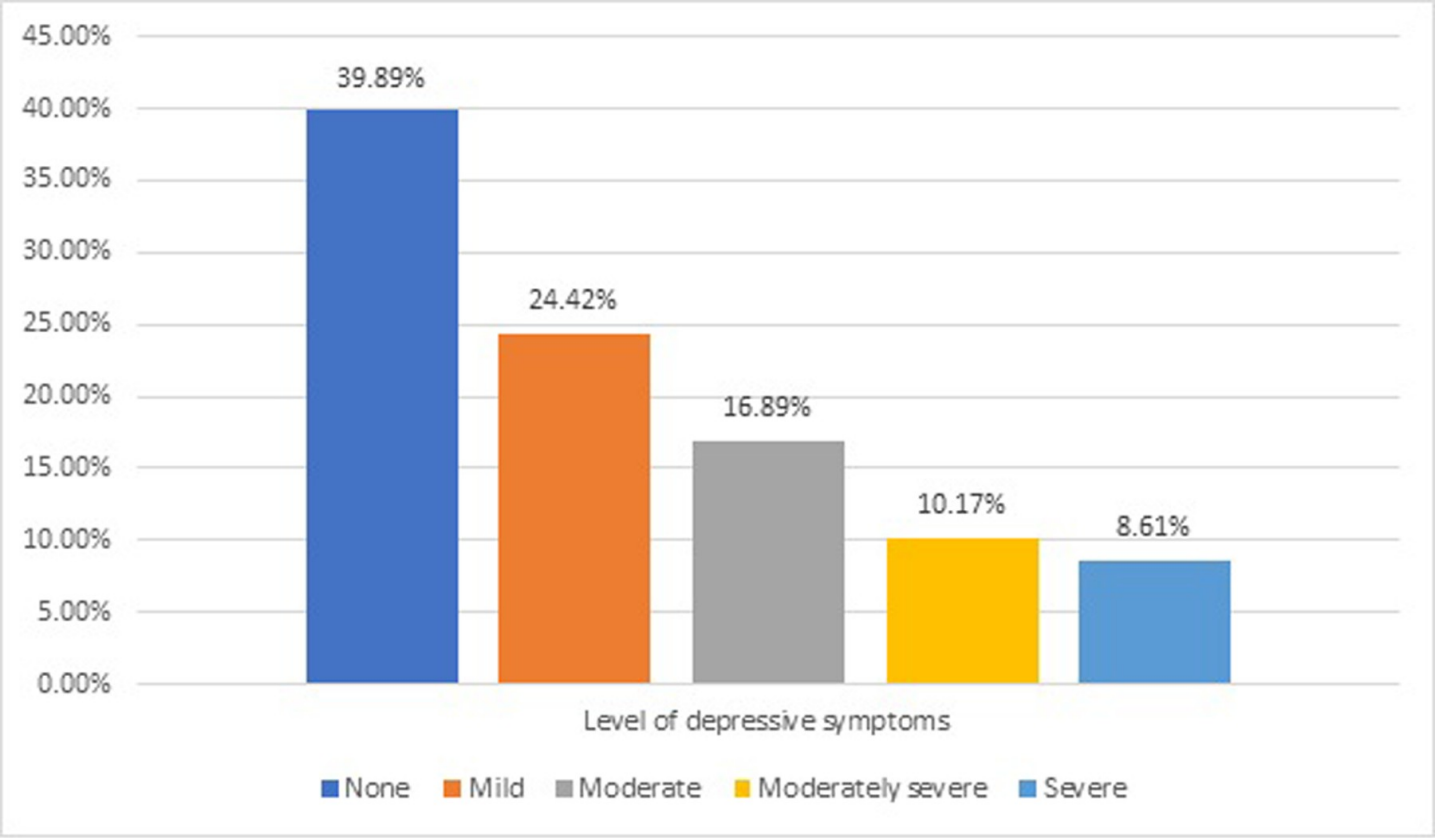
Prevalence of depressive symptoms according to PHQ-9 scores.

The prevalence of depressive symptoms of any severity was found 60.11%, with the prevalence of depressive symptoms decreasing with increasing severity. The cumulative prevalence of depressive symptoms of at least moderate severity was 35.67%. The prevalence of depressive symptoms according to PHQ-9 scores is given in Fig 2.

**Fig 3:**
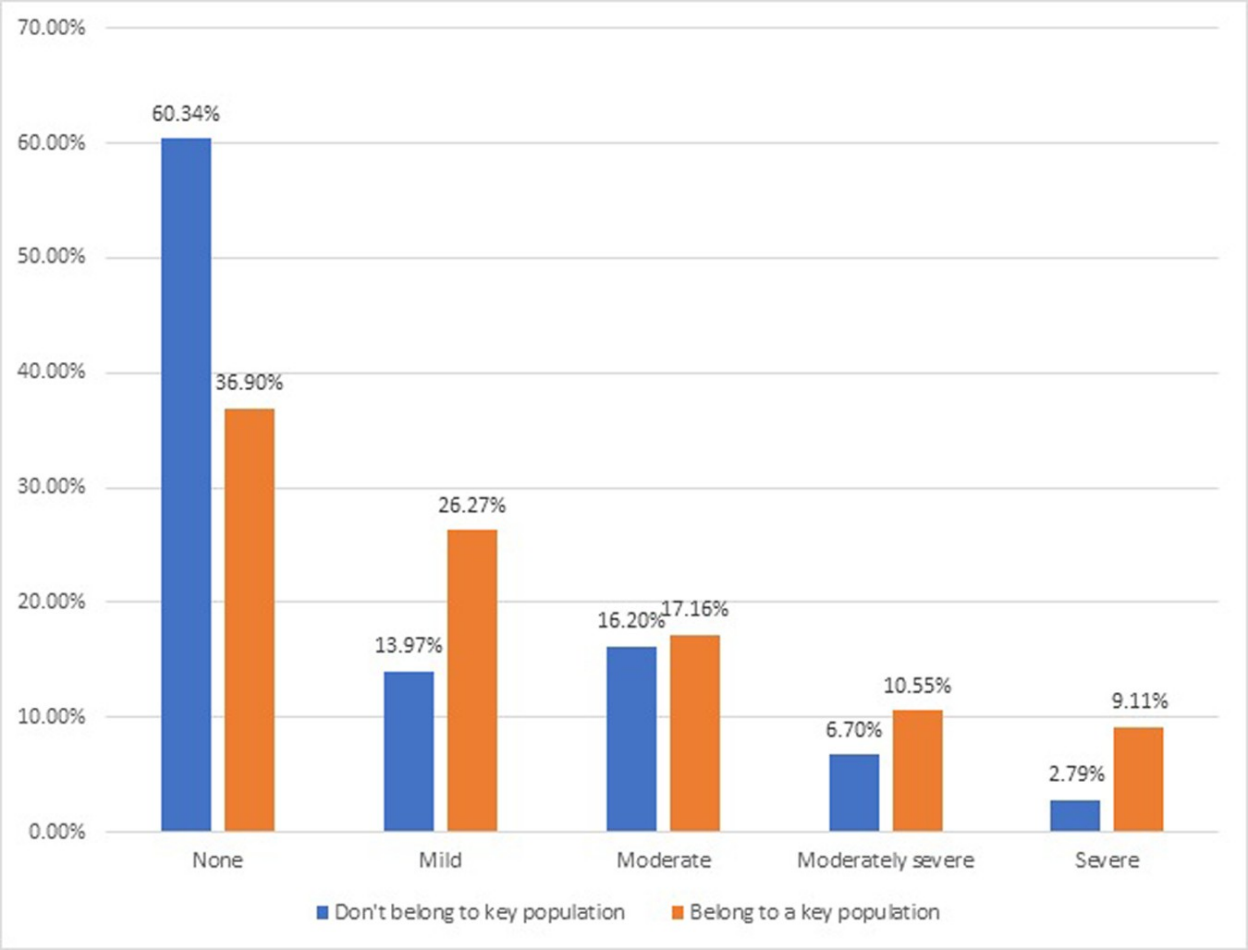
Prevalence of depressive symptoms among PLHIV from general population compared to those from the key population.

The prevalence of depressive symptoms was also computed to determine whether PLHIV who belong to any key population (MSM, sex worker, TG, person who use substance for recreation) had more depressive symptoms than the general population. The prevalence of depressive symptoms was higher in PLHIV from key populations that in PLHIV from the general population in all the categories. The results are shown in Fig 3.

We conducted a bivariate correlation analysis between PHQ-9 scores and sociodemographic factors. Factors with a correlation coefficient of 0.1 or more (regardless of the direction of correlation) were included in the step-wise multiple linear regression. The final model included all six predicting variables entered and predicted 18.2% of variance (adjusted R square = 0.182 p < .05) as shown in Table 2. The Durbin Watson test had a value of 1.839, thus supporting independence of observation assumption. The predictors included in the final model had tolerance value more than 0.1 and a variance inflated factor (VIF) value of less than 10. Thus, there was no multicollinearity between predictive factors included in the model. The normality of the residual assumption was also checked and was found valid. The model summary for stepwise multiple regression is presented in Table 2.

**Table 2:** Summary statistics of models generated through stepwise multiple linear regression.

Model	R	R-square	Adjusted R square	F change	Sig. F Change
Model 1. Predictors: (Constant), Being worried to meet life needs such as food and shelter in last 12 months	.279	.078	.077	6.888	.000
Model 2. Predictors: (Constant), Being worried to meet life needs such as food and shelter in last 12 months, female sex	.351	.123	.122	6.718	.000
Model 3. Predictors: (Constant), Being worried to meet life needs such as food and shelter in last 12 months, female sex, substance use	.405	.164	.162	6.563	.000
Model 4. Predictors: (Constant), Being worried to meet life needs such as food and shelter in last 12 months, female sex, substance use, being employed	.415	.172	.170	6.532	.000
Model 5. Predictors: (Constant), Being worried to meet life needs such as food and shelter in last 12 months, female sex, substance use, being employed, recruited through limited chain referral	.423	.179	.176	6.508	.001
Model 6. Predictors: (Constant), Being worried to meet life needs such as food and shelter in last 12 months, female sex, substance use, being employed, recruited through limited chain referral, doing sex work	.427	.182	.179	6.497	.015

In the final model, being worried to meet basic life needs such as food and shelter in last 12 months (95% confidence interval 3.98–5.68, p < .01), female sex (95% CI 2.703–4.49, p < .01), substance use (95% CI 2.379–3.88, p < .01), being employed (95% CI -2.36 to -.88, p < .01), being recruited through limited chain referral (95% CI -3.41 to .88, p< .01), and doing sex work (95% CI .225–2.061, p < .01) were significant predictors of depressive symptoms, as shown in Table 3.

**Table 3:** Predictors of depressive symptoms among PLHIV in Pakistan.

Serial no.	Variable	Unstandardized co-efficient		t	P value	95% confidence interval	
		B	Standard error			Upper bound	Lower bound
**1.**	**Intercept**	4.817	.443	10.872	.000	3.948	5.687
**2.**	**Being worried to meet life needs such as food and shelter in last 12 months**	2.188	.237	9.244	.000	1.724	2.653
**3.**	**Female sex**	3.599	.457	7.882	.000	2.703	4.495
**4.**	**Substance use**	3.133	.384	8.152	.000	2.379	3.887
**5.**	**Being employed**	-1.627	.378	-4.309	.000	-2.368	-.887
**6.**	**Recruited through limited chain referral**	-2.147	.645	-3.330	.001	-3.412	-.882
**7.**	**Doing sex work**	1.143	.468	2.443	.015	.225	2.061

Recruitment from a limited chain referral was negatively associated with depressive symptoms. We explored whether the stigma faced in an HIV-specific healthcare setting moderated the relationship between participant recruitment methods. Overall, the model significantly predicted depressive symptoms (F (3, 1484) = 12.31 P < .000). The model predicted only 2.43% of the variance (Adjusted R square = 0.0243 p < .000). The interaction between the participant recruitment method and stigma in the HIV-specific healthcare setting explained 0.3% of the variance (R square change of .0030 (p < .05). Th results are shown in Table 4.

**Table 4:** Moderator effect of the healthcare stigma in HIV-specific settings on recruitment strategy and depressive symptoms.

Serial no.	Variable	Unstandardized		t	P value	95% confidence interval	
		B	Standard error			Upper bound	Lower bound
1.	Intercept	8.0402	.2245	35.8218	.000	7.6000	8.4800
2.	Participant recruitment method (Reference HIV service delivery site)	-4.4897	.7830	-5.7342	.000	-6.0255	-2.9538
3.	Stigma while accessing health services for HIV	.04571	.4301	1.0627	.2881	-.3866	1.3008
4.	Participant recruitment method x Stigma while accessing health services for HIV	3.205	1.5050	2.1292	.0334	.2523	6.157

**Fig 4:**
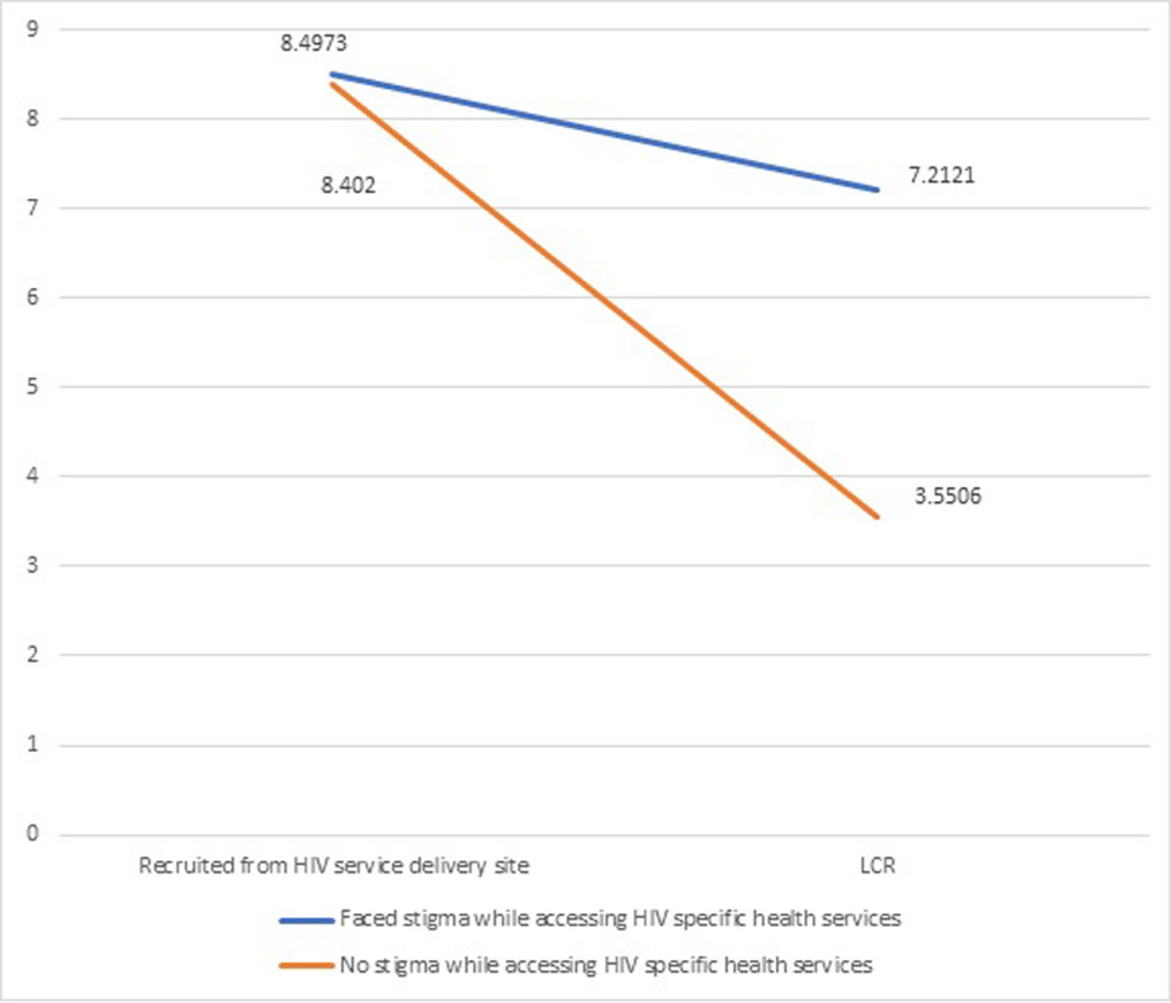
Plot of interaction between participant recruitment strategy and the stigma faced while accessing HIV-specific health services.

Fig 4 shows interaction between participant recruitment strategy and stigma faced while accessing HIV-specific health services (y-axis PHQ-9 score).

## Discussion

This is the first multicenter analysis on prevalence and predictors of depressive symptoms among PLHIV in Pakistan. According to the results of this study, 60.11% had depressive symptoms of any severity whereas the prevalence of at least moderate severity was 35.67%. Overall, the model only moderately explained the variance in depressive symptoms. Previously, the frequency of depressive symptoms was studied by Junaid et al. among PLHIV in Lahore by recruiting PLHIV from a single HIV treatment center and screening using PHQ-9. According to this study, the prevalence of depressive symptoms of moderate severity was 32.2%, which is very close to estimates from our study [14]. In another study by Ahmed et al. the prevalence of depressive symptoms based on the Hospital Anxiety and Depression Scale was 89.9%. About 80.3% of the sample in the stated study had comorbid anxiety and depressive symptoms [15]. It should be noted that in our study, only PLHIV who knew their status for at least one year were interviewed. In addition, our sampling strategy differed from that of studies mentioned above. The global prevalence of depression among PLHIV varies from 28% to 34% [21]. A recently conducted meta-analysis from China reported that the prevalence of depression among PLHIV is approximately 50.8% [22]. Another study from Nepal by Shrestha et al. concluded that the prevalence of depression among those seeking HIV treatment was 26.8%. In the study, about 84% of the study subjects were diagnosed with HIV more than 12 months ago [23]. A multinational study from six Asian Pacific countries concluded a 48% prevalence of depression among PLHIV [24]. A study from Africa reported that the prevalence of depression in their study participants was 28% [25].

In terms of the severity of depression, Junaid et al., reported that 18.9% of the study participants had moderate depressive symptoms, which was similar to our estimate of 16.89%. In our study, the prevalence of moderately severe depressive symptoms was almost twice that reported by Junaid et al. which is 10.17% versus 5.69%, whereas the prevalence of severe depressive symptoms was 8.61% in our sample compared with 7.59% reported by Junaid et al. [14] Our findings on the prevalence of moderate depressive symptoms are similar to those of a study from Asian-Pacific countries which reported a 12% prevalence of moderate depressive symptoms. However, in the same study, there was 5% prevalence of moderately severe depressive symptoms and 2% prevalence of severe depressive symptoms, which much low than those found in our study [24].

Disaggregating data on the prevalence of depressive symptoms among those who belong to any key population versus the general population shows that depressive symptoms across the severity spectrum were much more prevalent among PLHIV from key populations than among PLHIV from the general population. Mild depressive symptoms were twice as common among PLHIV from the key population, while moderate were almost equally present in both groups. However, moderately severe and severe depressive symptoms were almost 2–3 times more common among PLHIV from the key populations than in the general population. These group differences based on key population status have not reported in other studies from Pakistan. As stated above, these key populations face stigma and are criminalized in the country. Thus, minority stigma and discrimination add to day-to-day stressors which may translate to depressive symptoms. Since the current study is only a screening tool for depressive symptoms, it is not clear that among those who use substance, how much percentage of depressive symptoms are secondary to substance use and how many can be accounted as primary mood or depressive disorders.

In terms of the predictors of depressive symptoms, the factor that explained most of the variance was being worried to meet daily life needs, such as food and shelter, followed by female sex. However, employment conferred some protection against depressive symptoms. A hospital-based survey from Southeast Ethiopia concluded that a low level of income increased the odds of depression among PLHIV by 3.1. In the same study, stigma was the single most dominant predictor of depression [26]. Rabeya et al., have conducted a study to screen for depressive symptoms among PLHIV using the Beck Depression Inventory (BDI) in Bangladesh. The results of the study show that an increase in income reduced the odds of depressive symptoms among PLHIV [27].

Ahmed et al. have concluded that illicit substance use increased odds of depression by 1.87 whereas Junaid et al. report that odds of developing depressive symptoms 4.14 among those with illicit substance use [14, 15]. PLHIV with low or moderate social support were more likely to have depressive symptoms than to those with high social support [15]. Wedajo et al. have explored the role of social support and perceived stigma in predicting depressive symptoms in Northeast Ethiopia. The results of this study show that social support has a direct protective effect against depressive symptoms [28]. In other studies from Pakistan, male PLHIVs were reported more likely to have depressive symptoms than female PLHIV while in our study female sex was associated with depressive symptoms, with a difference of 3.5 points on PHQ-9 as compared to male PLHIV [15]. Apart from illicit drug use, sex work was also associated with increased depressive symptoms.

In our study, those recruited from limited chain referrals had fewer depressive symptoms than those recruited from HIV service delivery sites. Moderation analysis showed that while stigma faced while accessing HIV health services did not predict depressive symptoms, it moderated the relationship between depressive symptoms and participant recruitment strategies. The moderation analysis highlights that those who were recruited through limited chain referral and faced healthcare stigma had more depressive symptoms than those who did not face healthcare stigma while accessing HIV-specific services. Junaid et al. reported stigma increased odds of depressive symptoms by 3.505 whereas Ahmed et al reported stigma increased odds of depressive symptoms by 2.48 [14, 15].

Thus, it can be hypothesized that the stigma faced in healthcare settings and depressive symptoms may lead PLHIV to drop out from availing HIV services. It should be noted that the Global AIDS Strategy aims at 95-95-95 by 2025, which means 95% of PLHIV know their HIV status, of which 95% are on HIV treatment and 95% of these have undetectable viral load [29]. However, in Pakistan only 20.84% PLHIV are aware of their HIV status, of these 61.11% on HIV treatment and only 11.95% are virally suppressed [4]. Interventions to decrease healthcare stigma and addressing depressive symptoms among PLHIVs may improve treatment adherence and engagement among PLHIV [30].

A global modeling exercise was conducted in 2019, which showed that investing in mental health integration is a cost-effective intervention and each US dollar spent in treating mental health conditions in PLHIV saves 6.40 US dollars. This is higher than cost-effectiveness of treatment of mental health conditions elsewhere where 5 US dollars are saved per US dollar investment [31]. Additionally, the integration of mental health and HIV can avert 0.9 million new infections by 20230, partially mediated through improved medication adherence, suppressed viral load and decreased transmission [31]. Thus, investing in mental health of PLHIV is an effective intervention that must be considered in the HIV response in Pakistan.

The results of this analysis are based on the largest PLHIV survey conducted in Pakistan. However, these results should be cautiously interpreted. First, PHQ-9 is a screening self-reported tool for depressive symptoms and any screened person on PHQ-9 should be confirmed by trained professionals through clinical interview [32]. Thus, while we report the prevalence of depressive symptoms, the prevalence of depressive disorders can only be ascertained by including structured clinical interview schedules. In addition, we used purposive sampling and recruited most study participants from HIV service delivery sites. The inclusion criteria included people aged 18 years and above; and thus, the findings cannot be generalized to children and young adults aged less than 18 years. Furthermore, a significant proportion of PLHIV reside in the areas of the outbreaks. These communities may have unique characteristics and a different prevalence of depressive symptoms than in our sample. Lastly, since the study was a cross-sectional in design, causality cannot be established that is if the study participants developed depressive symptoms after the diagnosis of HIV or had these symptoms prior to it. This is especially important among PLHIV from key populations who have lived a considerable part of their lives in a highly stigmatized environment.

## Conclusion

There is a high prevalence of depressive symptoms among PLHIV in Pakistan. Inability to meet basic life needs, female sex, substance use, employment and facing stigma in a healthcare setting were predictors of depressive symptoms. Similarly, stigma faced in a healthcare setting moderates the relationship with depressive symptoms. Employment and the ability to meet life needs confer some protection against depressive symptoms. There is a need for socioeconomic empowerment, stigma reduction in healthcare settings and a robust screening program for depressive symptoms in the PLHIV community in the country.
